# Improving the Antioxidant and Anti-Inflammatory Activity of Fermented Milks with Exopolysaccharides-Producing *Lactiplantibacillus plantarum* Strains

**DOI:** 10.3390/foods13111663

**Published:** 2024-05-25

**Authors:** Roberta Prete, Francesca Dell’Orco, Giusi Sabatini, Federica Montagano, Natalia Battista, Aldo Corsetti

**Affiliations:** Department of Bioscience and Technology for Food, Agriculture and Environment, University of Teramo, 64100 Teramo, Italy; fdellorco@unite.it (F.D.); gsabatini@unite.it (G.S.); fmontagano@unite.it (F.M.); nbattista@unite.it (N.B.); acorsetti@unite.it (A.C.)

**Keywords:** exopolysaccharides, *Lactiplantibacillus plantarum*, fermented milks, antioxidant activity, ROS modulation, intestinal model

## Abstract

Exopolysaccharides (EPSs) producing lactic acid bacteria have been claimed to confer various health benefits to the host, including the ability to face oxidative and inflammatory-related stress. This study investigated the ability of food-borne *Lactiplantibacillus* (*Lpb.*) *plantarum* to improve the antioxidant activity of fermented milks by producing EPSs. Two *Lpb. plantarum* strains, selected as lower and higher EPSs producers, have been applied in lab-scale fermented milk production, in combination with conventional starters. Antioxidant activity was investigated in vitro using DPPH (1,1-diphenyl-2-picrylhydrazyl), ABTS (2,2-azino-bis(3-ethylbenzothiazoline-6-sulfonic acid), and FRAP (ferric reducing antioxidant power) assays while the ability to modulate reactive oxygen species (ROS) level was evaluated in an intestinal healthy model, subjected to both oxidative and inflammatory stress. Furthermore, to verify whether digestion affects functionality, fermented milks were evaluated before and after in vitro-simulated INFOGEST digestion. The results showed an improved antioxidant activity of fermented milk enriched with *Lpb. plantarum* LT100, the highest EPSs producer. Furthermore, the data showed a different ROS modulation with a protective anti-inflammatory effect of samples enriched with *Lpb. plantarum* strains. Our data suggest the use of selected EPS-producing strains of *Lpb. plantarum* as a natural strategy to enrich the functionality of fermented milks in terms of ROS modulation and inflammatory-related stress.

## 1. Introduction

Fermented foods have been playing an important role in human evolution. They have been appreciated for their sensory properties, shelf life, and safety but also for the presence of microbial metabolic products with several biological actions. The microbial metabolic activity during the fermentation process also improves the nutritional and functional properties of food with beneficial effects on human health [1]. Dairy products, especially yogurt and fermented milks, are widely consumed fermented foods produced by selected starter cultures, formally *Lactobacillus delbrueckii* subsp. *bulgaricus* and *Streptococcus thermophilus*, according to the Codex Alimentarius Standard No. 243/2003 [2], that received the EFSA (European Food Safety Authority) health claim for their role in improving lactose digestion [3]. Due to the presence of several bioactive compounds (e.g., peptides, minerals, vitamins), yogurt and fermented milk intake have been associated with gastrointestinal health via interaction with the immune system, with gut microbiota modulation and intestinal barrier homeostasis leading to a reduction in circulating biomarkers of chronic inflammation [4,5]. However, in order to enhance their functionality, yogurt and fermented milks can be enriched with the addition of probiotics [6]. Among them, lactic acid bacteria (LAB) and Bifidobacteria are widely used in dairy products to improve health benefits through production during the fermentation process of several beneficial compounds, such as bacteriocins, bioactive peptides, neurotransmitters, and exopolysaccharides (EPSs) [7]. From a technological perspective, EPSs are natural bio-thickeners, and EPSs in situ production by LAB is a viable alternative to artificial stabilizing thickeners/additives; thus, the use of EPS-producing strains in the production of dairy products is mainly applied to naturally improve food consistency and texture [8]. Besides that, EPSs produced by LAB have also gained attention for their correlation with microbe–host interactions and the microbial modulation of the immune system, as well as other beneficial effects such as antioxidant and anti-inflammatory activities [9,10]. Moreover, EPSs also play a role in helping bacteria to endure gastrointestinal stress, leading to a longer persistence of bacteria in the gut, and likely better health effects on the host [11]. Nowadays, due to the increasing accumulation of reactive oxygen species (ROS) that induce in vivo oxidative stress, strictly related to many chronic inflammatory conditions in the gut, antioxidative strategies from natural sources, including LAB-derived EPSs, have been investigated as dietary interventions to address ROS overproduction and accumulation [12]. As other beneficial features, diverse factors can influence the bacterial capability to produce EPSs; thus, the type of EPS, the amount of EPS production, and the effective EPS health benefits are widely species- and strain-specific [8]. Among LAB species, *Lactiplantibacillus* (*Lpb.*) *plantarum* is a flexible species with a long history in fermentation as starter cultures, and many strains have been used as adjunct probiotic cultures, showing to be a naturally safe and efficient strategy in preventing and triggering various diseases via the amelioration of oxidative stress and inflammation. Moreover, it has been shown that different *Lpb. plantarum* strains can exert these beneficial effects via the production of EPSs [13]. In addition, there is a body of in vitro evidence showing that EPSs isolated from different food-associated *Lpb. plantarum* strains possess antioxidant activity [14,15,16,17,18]. Recently, some in vivo studies confirmed the antioxidative role of EPSs produced by LAB species. For instance, the administration of EPSs from *Lpb. plantarum* YW11 and *Lactobacillus helveticus* KLDS1.8701 alleviated oxidative stress in aging mouse models [19,20]. These studies reported and suggested the application of isolated and purified EPSs produced by different LAB as a healthy and “green” strategy to counteract oxidative stress and inflammation; however, an alternative strategy to achieve a similar outcome could be the application of selected EPS-producing LAB strains for dairy product fortification by the in situ release of EPSs with ROS modulation and anti-inflammatory activity during the fermentation process.

**Figure 1:**
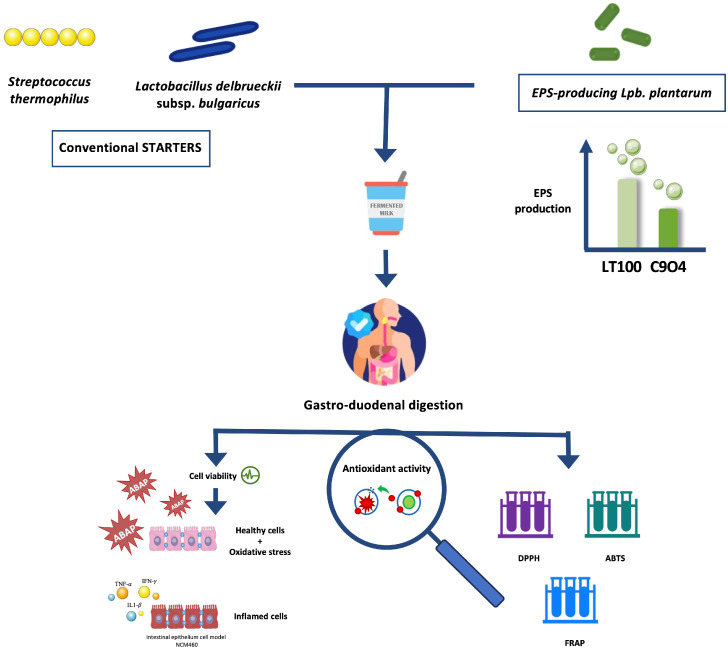
Graphical scheme of the experimental design of this study. Graphical illustrations were created by using some graphical elements from Servier Medical Art by Servier, available on https://smart.servier.com/ (accessed on 24 November 2021) under a Creative Commons Attribution 3.0 Unported License.

Based on that, the aim of this study was to investigate the ability of some food-borne *Lpb. plantarum* to enhance the antioxidant activity of fermented milks by producing EPSs during the fermentation process, using an innovative approach that combines the in vitro screening and evaluation of *Lpb. plantarum* strains to be selected for the lab-scale fermentation process with the evaluation of the anti-inflammatory activity of *Lpb. plantarum*-enriched fermented milks via ROS modulation by using an inflamed intestinal model. Firstly, *Lpb. plantarum* strains isolated from fermented foods were selected based on their EPS production in fermented milks, and then the antioxidant activity of EPSs produced during fermentation was investigated in vitro. Furthermore, ROS modulation by fermented milks containing EPSs was evaluated in an intestinal epithelium model derived from the colonic mucosa of a healthy individual (NCM460), in which both oxidative and inflammatory stresses were induced. The putative impact of the digestion process on the biological activity of fermented milks was also considered. Figure 1 shows a graphical scheme of the experimental design of this study.

## 2. Materials and Methods

### 2.1. Bacteria Used in This Study

The *Lpb. plantarum* strains investigated in this study were selected among our laboratory collection at the University of Teramo (Table 1) for their ability to endure oxidative stress and their antioxidant and anti-inflammatory activity, previously reported [21]. All *Lpb. plantarum* strains were isolated from different fermented foods (table olives and raw-milk cheeses) and genetically and phenotypically characterized for several probiotic traits [22,23,24]. *Streptococcus thermophilus* (DSM20259) and *Lactobacillus delbrueckii* subsp. *bulgaricus* (DSM20081) were obtained from DSMZ—German Collection of Microorganisms and Cell Cultures and were included in this study as conventional starter cultures in lab-scale fermented milk production. All strains of *Lpb. plantarum*, *Lb. delbrueckii*, and *S. thermophilus* were cultured under microaerophilic conditions at 37 °C in MRS (de Man–Rogosa–Sharpe) and M17 broth, and they were subcultured overnight before each experiment.

### 2.2. Growth Compatibility Assay among Bacterial Strains

In order to use the selected strains as a mixture in lab-scale fermented milk production, an in vitro growth-inhibition assay (cross-compatibility test) was performed to evaluate the interaction among strains in terms of growth compatibility, according to Prete et al. [22]. The assay was performed by using agar plates containing the following media: MRS, M17, and PCA (Plate Count Agar). Briefly, 10 µL from each overnight culture was inoculated vertically for the donor strain and horizontally for the test strains; the presence of inhibition zones in the overlapping area means strain incompatibility.

### 2.3. Ruthenium Red Assay

All the strains were preliminarily screened for their EPSs production on Ruthenium Red (RR; 0.08 g/L) agar plates containing M17, MRS, and PCA with different carbon sources (glucose, lactose, and sucrose), as reported in Table S1. Overnight cultures were streaked on the respective RR agar plates, and after incubation at 37 °C for 48 h, white or pink colonies indicate EPS+ or EPS− strain behavior, respectively [25].

### 2.4. EPS Production in Single-Inoculated Fermented Milks

In order to select the lowest and the highest EPS-producing strains, the ability of *Lpb. plantarum* strains to produce EPSs in fermented milks was assessed. Milk fermentation was carried out using partially skimmed UHT milk, from TreValli Swan Milk, Italy, purchased from the supermarket, with the following composition: proteins: 3.2%, fats: 1.6%, and carbohydrates: 4.9%. The sterilized and cooled UHT milk was subjected to single inoculation with each *Lpb. plantarum* (LT100, LT52, C9O4, and O13), *S. thermophilus* (DSM20259), and *Lb. delbrueckii* subsp. *bulgaricus* (DSM20081), followed by incubation at 37 °C and 42° (for *Lb. delbrueckii* subsp. *bulgaricus*) for 48 h. The fermentation was stopped by storing the samples at 4 °C when the pH reached 4.55–4.65. The total EPSs extraction was performed according to the protocol described by Ayyash et al. [18] with minor modifications. Briefly, fermented milks were first heated in boiling water (90–95 °C) for 10 min to inactivate any endogenous enzymes, and then centrifuged at 4000 rpm for 10 min to isolate bacteria cells and coagulated proteins. Trichloroacetic acid (TCA) stock solution (85%) was then added to the supernatant. Subsequently, centrifugation at 14,000 rpm for 20 min at 4 °C was carried out to further precipitate the remaining proteins. The collected supernatants were then combined with three volumes of chilled absolute ethanol to precipitate EPSs, and centrifuged again at 13,000 rpm for 20 min at 4 °C. The obtained pellets were dissolved in deionized distilled water. The phenol–sulfuric acid method [26] was used to quantify the crude EPSs content. Briefly, 1 mL of EPS solution was combined with 0.5 mL of 5% aqueous phenol solution. Following that, 2.5 mL of concentrated sulfuric acid was quickly added to the mixture and incubated in ice for 10 min to allow the color development, measured at 490 nm by using a spectrophotometer (DU530 UV/Vis spectrophotometer, Beckman Coulter, Irvine, CA, USA). Deionized distilled water was used as the blank. Residual milk sugars were also calculated from fresh milk and subtracted from all data. The crude EPSs content of each sample was then calculated by using a glucose (50–750 mg/L) standard curve (R^2^ = 0.998, Figure S1), and the results, from three different replicates, are expressed as mg/L glucose equivalent.

### 2.5. Lab-Scale Fermented Milk Production

Fermented milks were prepared as previously described with some modifications [27,28,29,30]. Partially skimmed milk, described above, was inoculated by different LAB and then incubated at 42 °C until the pH reached 4.55–4.65; the milk was then stored at 4 °C overnight. As reported in Table 2, the experimental plan involved 4 different samples fermented by different LAB as follows: (1) co-inoculation with *S. thermophilus* DSM20259 and *Lb. delbrueckii* subsp. *bulgaricus* DSM20081, as in conventional yogurt (3% *v*/*v* of each starter); (2) *S. thermophilus* DSM20259, *Lb. delbrueckii* subsp. *bulgaricus* DSM20081, and *Lpb. plantarum* LT100 as a high EPS producer (3% *v*/*v* of each starter and 5% LT100); (3) *S. thermophilus* DSM20259, *Lb. delbrueckii* subsp. *bulgaricus* DSM20081, and *Lpb. plantarum* C9O4 as a low EPS producer (3% *v*/*v* of each starter and 5% C9O4); and (4) a commercial yogurt (Sterzing-Vipiteno).

### 2.6. Monitoring of Fermentation

Fermentation was followed by physicochemical and microbiological analysis. The pH value of fermented products was estimated during fermentation every 1 h, using a pH meter (pH80 + DHS, XS Instruments, Carpi, Italy) from 6.8 to 3.4. Titratable acidity was measured by titrating 10 g of the samples blended with 10 mL of distilled water using 0.1 N NaOH solution until pH 8.3. Titratable acidity was calculated as the percent of lactic acid, as described by Chang et al. [30]. Furthermore, after fermentation, the viability of all strains inoculated was checked by the viable plate count in MRS or M17 agar plates. Then, all samples were stored overnight at 4 °C for further analysis [31].

### 2.7. EPS Quantification in Co-Inoculated Fermented Milks

The EPSs released during fermented milk production by different inoculated LAB strains were quantified using the phenol–sulfuric acid method, as already described above.

### 2.8. Gastro-Duodenal Simulated INFOGEST Digestion

The simulated digestion process was performed by following the standardized in vitro INFOGEST protocol [32]. Briefly, 5 g of each sample was sequentially subjected to simulated salivary fluid (with 75 U/mL of α-amylase) for 2 min, simulated gastric fluid (with 25,000 U/mL of pepsin) at pH 3 for 2 h, and simulated intestinal fluid (with 12 g/L of bile salts and 2 g/L of pancreatin) at pH 7 for 2 h. All digestion steps were performed at 37 °C under shaking to mimic gastrointestinal peristalsis. Digested samples were centrifuged at 4000 rpm for 15 min, filtered with 0.22 μm syringe filters, and then stored at −20 °C until use.

### 2.9. In Vitro Antioxidant Activity

In order to evaluate the antioxidant activity of EPSs produced during fermentation, aqueous extract of each sample was prepared as reported by [33]. Briefly, all samples (10 g) mixed with 2.5 mL of deionized water, after adjusting the pH to 4.0 (with HCl 1 M), were incubated at 45 °C for 10 min, and then centrifuged (13,000 rpm, 20 min, 4 °C). Supernatants were recovered, and the pH was neutralized in each sample (with NaOH 1 M) and then centrifuged again (13,000 rpm, 20 min, 4 °C). Supernatants of each sample were collected and used for further analysis. Antioxidant activity was evaluated by performing three different microplate chemical assays, DPPH (1,1-diphenyl-2-picrylhydrazyl radical), ABTS ([2,2-azino-bis(3-ethylbenzothia-zoline-6-sulfonic acid)], and FRAP (ferric reducing antioxidant power), following the protocols described in detail by Prete et al. [21]. Moreover, the same assays were used to evaluate whether *S. thermophilus* (DSM20259) and *Lb. delbrueckii* subsp. *bulgaricus* (DSM20081), used as conventional starters, possess antioxidant properties in vitro, to exclude their potential impact on fermented samples.

### 2.10. Intestinal Epithelium Model

NCM460 cells (INCELL Corporation, LCC, Sant’Antonio, TX, USA) were used as the intestinal epithelium [24]. The cell line was grown in INCELL’s M3:Base medium supplemented with the addition of 10% (*v*/*v*) Fetal Bovine Serum (FBS, Corning, NY, USA), 1% (*v*/*v*) Penicillin–Streptomycin (Pen-Strep, Corning, NY, USA), and 1% (*v*/*v*) Non-Essential Amino Acids (NEAAs, Corning, NY, USA) and incubated at 37 °C in a 5% CO_2_ atmosphere. Cells were seeded (1.0 × 10^5^ cells/well in 96-well plates) for 24 h to achieve a confluent monolayer before each assay.

### 2.11. Cell Viability Assay

The colorimetric 3-(4,5-dimethylthiazole-2-yl)-2,5-phenyl tetrazolium bromide (MTT) assay, widely used for the detection of cell viability, was performed to confirm the non-cytotoxic effect of the fermented milks toward NCM460 human cells according to Garcia et al. [24], with slight modifications. Briefly, three different concentrations of aqueous extract from each fermented milks (5 μL/mL, 10 μL/mL, 20 μL/mL of medium) were added to NCM460 confluent monolayers, in order to assess possible cytotoxicity in a healthy intestinal model (i.e., without any oxidative or inflammatory stimulus). Subsequently, 96-well plates were incubated at 37 °C for 24 h in a 5% CO_2_ atmosphere. After incubation, the supernatants were removed and 10 μL of MTT (5 mg/mL) and 100 μL of DMEM were added to each well to allow incubation for 4 h at 37 °C in a 5% CO_2_ atmosphere. Then, the supernatants were removed and the formazan crystals were dissolved by adding 100 μL of acidified isopropanol followed by mechanical shaking. The OD from each well was measured at 570 nm and 630 nm by an EnSpire multimode plate reader (PerkinElmer, Waltham, MA, USA). Control wells (without samples) and blank wells (without cells) were also included to remove background noise and blank measurements. The cell viability percentage was calculated by means of the following equation: [OD of tested sample (NCM460 treated with fermented milks)]/[OD of control wells (untreated NCM460) × 100].

### 2.12. ROS Modulation Assessment on Intestinal Cell Models

To evaluate ROS modulation by digested fermented products, the fluorometric microplate dichlorofluorescein diacetate (DCFH-DA) assay described by Prete et al. [21] was performed. The impact of fermented milks on the release of ROS by intestinal cells was investigated upon the induction of both oxidative and inflammatory stress, as described as follows:

(i) NCM460 cells were treated for 24 h with digested samples, with the highest sample concentration exhibiting no cytotoxic effects (20 μL/mL of medium) in M3:Base medium with 1% FBS, at 37 °C in a 5% CO_2_ atmosphere. After two washing steps with Hanks’ Balanced Salt Solution (HBSS), NCM460 cells were incubated with 100 μL of DCF-DA (25 μM) for 1 h at 37 °C in a 5% CO_2_ atmosphere. Then, each well was washed twice with HBSS and oxidative stress was induced by adding 100 μL of 2,2′-azobis (2-amidinopropane) dihydrochloride solution, ABAP (600 μM), to promote ROS production.
(ii) NCM460 cells were treated for 24 h with digested samples, with the highest sample concentration exhibiting no cytotoxic effects (20 μL/mL medium) simultaneously with an inflammatory cocktail (10 ng/mL of TNF-α, 10 ng/mL of IFN-γ, and 5 ng/mL of IL1-β) in M3:Base medium with 1% FBS, at 37 °C in a 5% CO_2_ atmosphere, in order to mimic the inflammatory status of IBD [24]. Subsequently, two washing steps with HBBS were performed and inflamed-treated NCM460 cells were incubated with 100 μL of DCF-DA (25 μM) for 1 h at 37 °C in a 5 CO_2_ atmosphere.

Fluorescence emission was measured every 5 min for 1 h by an EnSpire Multimode Plate Reader (PerkinElmer, Waltham, MA, USA) at excitation and emission wavelengths of 485 and 535 nm, respectively. For each assay, HBSS fluorescence values were used as the blank, untreated NCM460 cells were used as the negative control, and NCM460 treated with ABAP and the inflammatory cocktail were used as the positive controls. The data are expressed as mean values of the percentage of fluorescence units with respect to fluorescence units measured for positive controls.

### 2.13. Statistical and Data Analysis

All data, obtained from three biological and three technical replicates, are reported as mean values ± SEM, and they were analyzed by means of PRISM 8.3.1 (GRAPHPAD Software Inc., La Jolla, CA, USA) using One-Way analysis of variance (ANOVA) followed by Tukey’s multiple comparisons analysis and by Student’s *t*-test with a level of *p* < 0.05 considered statistically significant.

## 3. Results

### 3.1. Growth Compatibility Assay among Bacterial Strains (Cross-Compatibility Test)

An in vitro growth assessment, namely the cross-compatibility test, was applied to evaluate the strains inter-isolate influence on growth [22]. Briefly, all the strains were benchmarked against each other to assess their own potential to act against others as displacement strains. The cross-compatibility test showed that all strains allowed the growth of each other over 48 h with no growth inhibition detected (Table S2). These data confirmed, in vitro, the suitability of both conventional starter and *Lpb. plantarum* strains to be used in a mixture. In mixed-culture fermentations, the combinations of different microbes can potentially influence the fermentative properties and microbial growth, which can affect bacterial community structures and functions [34]. From the perspective of using a mixed culture of conventional starters combined with health-promoting *Lpb. plantarum* strains to enhance the functionality of the final products, achieving a positive microbial interaction in terms of a lack of strain displacement is of paramount importance, indicating that the introduction of different species in the starter mixture may not alter the fermentation process.

### 3.2. EPS Production Plate Screening

**Figure 2:**
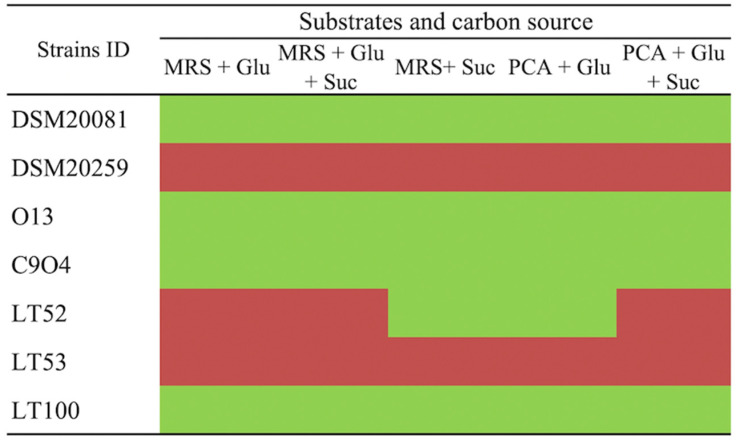
Ruthenium Red plate assay for EPS-producing screening. (Glu: glucose, Suc: sucrose) in different culture media. Green boxes refer to ropy (EPS-producers) and red boxes to non-ropy strains (non-EPS producers).

The putative EPSs production by conventional starters and *Lpb. plantarum* strains was evaluated in a plate screening by using the Ruthenium Red dye in different growth media. As reported in Figure 2, the EPSs production was evaluated by using media containing different carbon sources that allow the EPS-producer strains to be discriminated. Ruthenium Red stains the bacterial cell walls; thus, non-ropy species produce pink/red colonies growing on Ruthenium Red plates. EPSs prevents this staining in ropy strains, resulting in white colonies [25]. *Lpb. plantarum* O13, C9O4, and LT100 growth resulted in white colonies (ropy strains), which were considered positive for EPSs production, whilst *Lpb. plantarum* LT53 showed pink colonies (non-ropy strains) and was excluded from further analyses. A similar screening approach was successfully reported by other studies [25,35]. Lastly, *S. thermophilus* DSM20259 was a non-EPS producer whilst *Lb. delbrueckii* subsp. *bulgaricus* could be considered a slight EPS-producer strain.

### 3.3. EPSs Quantification in Single-Inoculated Fermented Milks

**Figure 3:**
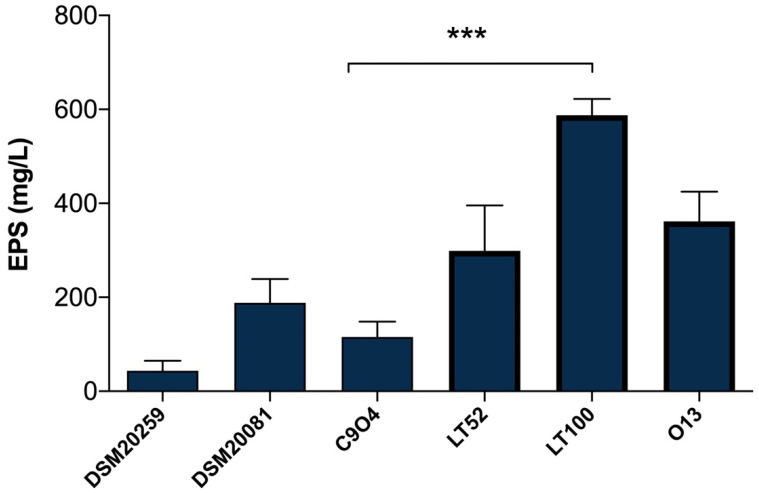
Quantification of EPSs production in milk samples fermented by DSM20259, *S. thermophilus*; DSM20081, *Lb. delbrueckii* subsp. *bulgaricus*; C9O4, *Lpb. plantarum*; LT52, *Lpb. plantarum*; LT100, *Lpb. plantarum;* and O13, *Lpb. plantarum*. Data are reported as mean values ± SEM. Statistical analysis was performed by One-Way ANOVA followed by Tukey’s multiple comparisons post hoc test (*** *p* = 0.0002).

EPSs quantification in *Lpb. plantarum*-enriched fermented milks was carried out with the phenol–sulfuric acid method [26], which is a simple, practical, and widely used method to quantify total polysaccharides in food samples. As shown in Figure 3, the results highlight a different amount of EPSs produced among *Lpb. plantarum* strains, allowing those with a higher and lower capability to produce EPSs to be selected. The EPSs production among *Lpb. plantarum* strains ranged from 115.55 (for C9O4) to 587.77 mg/L (for LT100) glucose equivalent (Figure 3); thus, C9O4 was selected as the lowest EPS producer and LT100 as the highest EPSs producer, among the tested strains. Moreover, the results of *S. thermopilus* DSM20259 and *Lb. delbrueckii* subsp. *bulgaricus* DSM20081 confirmed that the amount of EPSs production by conventional starters may have no impact on the final product, showing a minimal ability to produce EPSs compared to that of *Lpb. plantarum* strains that showed a greater EPS-producing ability in a strain-dependent manner, as also recently reported [36,37,38,39]. The capability to produce EPSs depends on several key factors influencing bacterial growth (e.g., temperature, incubation time, pH, oxygen rate, and carbon and nitrogen sources) and the amount of EPSs production is largely strain-specific [8]. Thus, the selection of EPS-producing strains provides an important tool to improve the technological and functional properties of dairy products [8].

### 3.4. Lab-Scale Fermented Milk Production: Physicochemical and Cell Viability Parameters

The fermented milk production was carried out as reported above at a temperature of 42 °C. The fermentation process was monitored through physicochemical analyses by monitoring pH and titratable acidity, to determine whether the addition of *Lpb. plantarum* strains could affect these parameters. After 24 h, at the end of fermentation, the viability of the fermenting LAB was assessed through viable plate counts on MRS and M17 agar plates. Table 3 reports microorganism viability, pH values, and the relative concentration of lactic acid (g/L) after 24 h of fermentation.

### 3.5. EPSs Quantification in Lpb. plantarum-Enriched Fermented Milks

**Figure 4:**
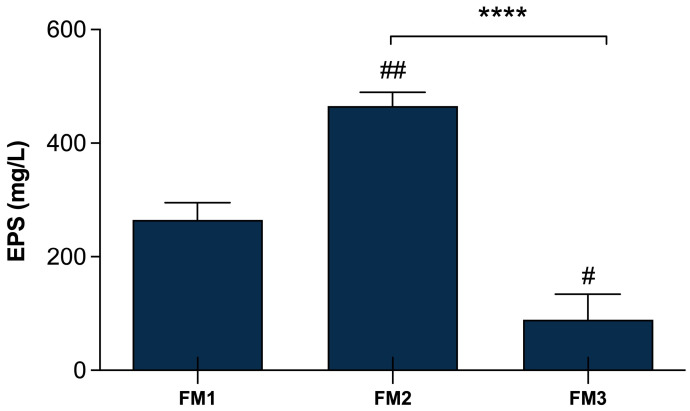
EPSs production in fermented milk samples. FM1 fermented by *S. thermophilus* DSM20259 and *L. delbrueckii* subsp. *bulgaricus* DSM20081; FM2 fermented by *S. thermophilus* DSM20259, *L. delbrueckii* subsp. *bulgaricus* DSM20081, and *Lpb. plantarum* LT100; FM3 fermented by *S. thermophilus* DSM20259, *L. delbrueckii* subsp. *bulgaricus* DSM20081, and *Lpb. plantarum* C9O4. Data are reported as mean values ± SEM and they are expressed as mg/L glucose equivalent. Statistical analyses were performed by One-Way ANOVA followed by Tukey’s multiple comparisons post hoc test (FM1 vs. FM2 ^##^ *p* < 0.005; FM1 vs. FM3 ^#^ *p*< 0.05; FM2 vs. FM3 **** *p* < 0.0001).

The quantification of EPSs production in the final products was performed in order to evaluate whether the addition of the two selected *Lpb. plantarum* strains can affect the EPSs amount in the different types of fermented milks, comparing them with a fermented milk produced only by the two conventional starters. Figure 4 shows significant differences (*p* < 0.0001) among FM2, produced by the fermentation of two conventional starters DSM20259 and DSM20081 with the addition of *Lpb. plantarum* LT100 compared to FM3 containing *Lpb. plantarum* C9O4, reflecting the major capability of LT100 to produce EPSs in the final product, as already observed in single-inoculated fermented milks (Figure 2). Moreover, FM3 produced by the fermentation of *Lpb. plantarum* C9O4 in combination with conventional starters results in the product with the lowest amount of EPSs, confirming the lower ability of C9O4 to produce EPSs as observed in single-inoculated fermentation (Figure 3). Furthermore, the production of EPSs produced by the starters does not influence the production by *Lpb. plantarum*.

### 3.6. In Vitro Antioxidant Activity

#### 3.6.1. In Vitro Antioxidant Activity of Conventional Starters and *Lpb. plantarum*

**Figure 5:**
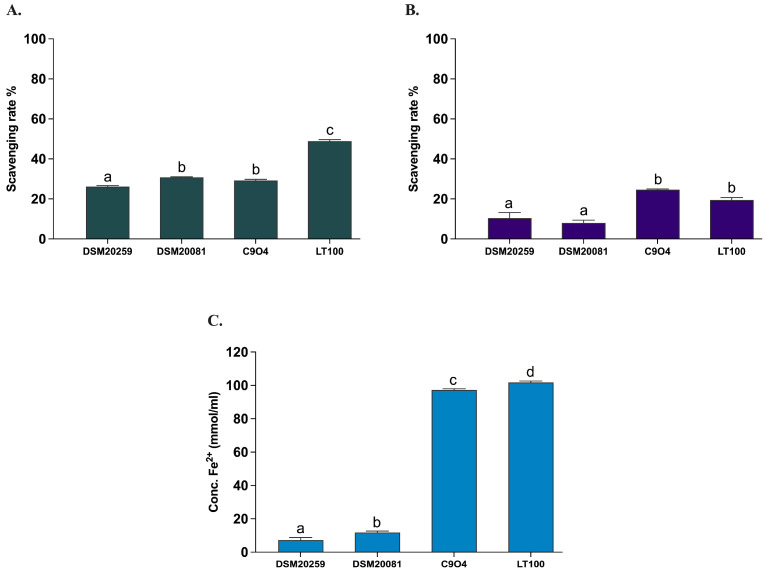
Antioxidant activity of conventional starters and *Lpb. plantarum* strains. (**A**) ABTS, (**B**) DPPH, (**C**) FRAP. Data are reported as mean values ± SEM and statistically analyzed by One-Way ANOVA followed by Tukey’s multiple comparisons test (different letters mean significant differences among samples, *p* < 0.05).

All *Lpb. plantarum* strains have been previously tested and selected for their antioxidant activity in vitro [21]. In this study, the antioxidant activity of the two conventional starter strains *S. thermophilus* and *Lb. delbruckeii* subsp. *bulgaricus* was evaluated by measuring the in vitro DPPH and ABTS radical scavenging activities, as well as the ferric iron reducing capacity (FRAP) according to Prete et al. [21]. *Lpb. plantarum* strains (C9O4 and LT100) were included in the assays to confirm the analysis previously carried out by Prete et al. [21]. As shown in Figure 5, the antioxidant capacity of the two conventional starters was minimal (DPPH < 10%, ABTS < 30%, and FRAP < 20 mmol Fe^2+^/mL) and significantly lower compared to that of the *Lpb. plantarum* strains, for which an ROS neutralization activity >20% for DPPH and >30% for ABTS and a FRAP >100 mmol Fe^2+^/mL were confirmed, as already reported [21].

#### 3.6.2. In Vitro Antioxidant Activity of Fermented Milks

**Figure 6:**
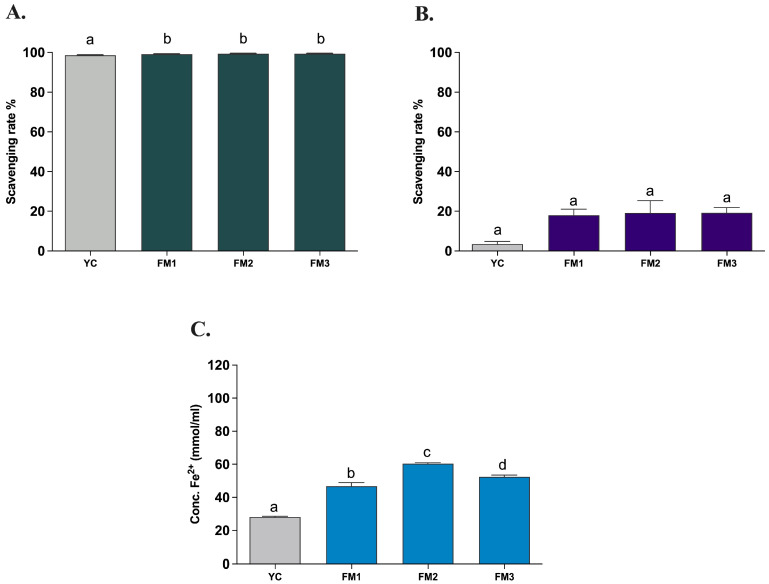
Antioxidant activity of fermented milks evaluated by (**A**) ABTS, (**B**) DPPH, and (**C**) FRAP assays. YC = commercial yogurt, FM1 = fermented milk produced by conventional starters, FM2 = fermented milk produced by conventional starters and *Lpb. plantarum* LT100 (high-EPS producer), FM3 = fermented milk produced by conventional starters and *Lpb. plantarum* C9O4 (low EPS producer). Data are reported as mean values ± SEM and statistically analyzed by One-Way ANOVA followed by Tukey’s multiple comparisons test (different letters mean significant differences among samples *p* < 0.0001).

The evaluation of the in vitro antioxidant activity of all samples was carried out after INFOGEST digestion, with the three different types of in vitro assays (ABTS, DPPH, and FRAP) used to assess the antioxidant properties of all strains. This allowed the potential functionality to be evaluated based on the amount of EPSs produced during fermentation, to compare the samples with a commercial yogurt (YC), and to verify the persistence of notable antioxidant activities after the simulated digestion process under in vivo conditions. The evaluation of the antioxidant activity determined through the in vitro ABTS method demonstrates a strong antioxidant capacity (>99%) of all samples also after the INFOGEST digestion protocol, showing a statistically significant difference (*p* < 0.0001) between FM1, FM2, and FM3 fermented milks compared to the commercial yogurt (YC) (Figure 6A). On the other hand, the evaluation of antioxidant activity using the DPPH method revealed a lower (<20%) scavenging activity, with no significant differences among samples (Figure 6B). The decrease in the scavenging activity could be explained by the nature of the reaction environment (alcoholic solution) as EPS is a hydrophilic molecule and therefore soluble in an aqueous environment. A lower antioxidant activity of hydrophilic compounds using DPPH has already been reported by other studies [16,40,41]. Finally, investigating the ferric reducing antioxidant power, the FRAP assay showed a good iron-reducing capacity of fermented milk samples (Figure 6C). In particular, FM2 and FM3 (enriched with *Lpb. plantarum* strains) showed a higher electron donation capacity (60.37 ± 1.00 and 52.37 ± 1.58 mmol/mL, respectively) with significant differences (*p* < 0.0001) compared to commercial yogurt (28.14 ± 0.70 mmol/mL) and to that containing only the two conventional starters (FM1, 46.81 ± 3.60 mmol/mL), in agreement with the results of ABTS. Furthermore, sample FM2, fermented with LT100, has a statistically significant (*p* < 0.0001) and greater reducing capacity than sample FM3, fermented with C9O4, in accordance with the different ability to produce and release EPSs in the final product.

### 3.7. Evaluation of the Impact of Fermented Milks on Intestinal Model

#### 3.7.1. Non-Cytotoxicity of Fermented Milks (MTT Assay)

**Figure 7:**
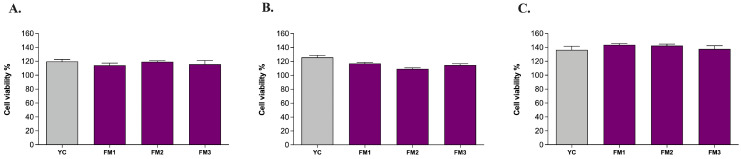
Evaluation of the viability of human intestinal cells incubated with digested fermented milks at different concentrations: (**A**) 5 μL/mL, (**B**) 10 μL/mL, and (**C**) 20 μL/mL. Grey columns represent commercial yogurt (YC) while purple columns represent fermented milks obtained in thi study (FM1, FM2, FM3). Data are reported as mean values ± SEM and they were statistically analyzed using One-Way ANOVA followed by Tukey’s multiple comparisons test (*p* > 0.05).

In order to confirm the absence of any cytotoxic effect of all samples upon ingestion, the MTT colorimetric assay was performed on human intestinal cells (NCM460) in healthy conditions (i.e., without any oxidative or inflammatory stimulus) according to Garcia et al. [24]. The results revealed that all samples are likely to leave the cellular metabolic activity of healthy NCM460 cells unchanged or even improved with no significant differences among samples (Figure 7). Furthermore, comparing the results of digested samples with those of undigested samples (Figure S2), there are no significant differences induced by the digestion step, as well as among the different concentrations investigated (5 μL/mL, 10 μL/mL, 20 μL/mL). Based on that, the highest concentration with no cytotoxic effect (20 μL/mL) was chosen for further analysis on ROS modulation.

#### 3.7.2. Impact of Fermented Milks on ROS Modulation in Intestinal Models

**Figure 8:**
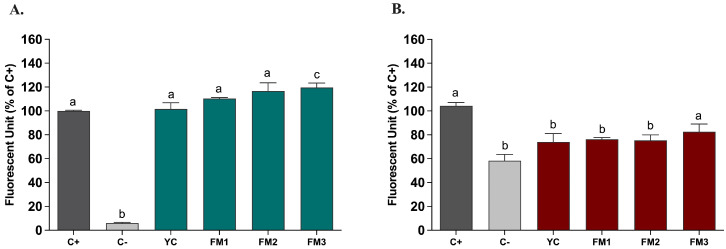
ROS modulation by digested fermented milks (20 μL/mL) assessed in intestinal models of (**A**) induced oxidative stress and (**B**) induced inflammation. Dark grey columns represent positive control, light grey columns represent negative control while green and red columns represent cells treated with yogurt and fermented milk samples. Data are reported as mean values ± SEM and statistically analyzed using One-Way ANOVA followed by Tukey’s multiple comparisons test (different letters mean significant differences among samples, *p* < 0.05).

Based on the in vitro evidence regarding the enhanced ability to partially neutralize ROS, we tested the potential protective impact of digested samples (20 μL/mL) on ROS modulation, by applying the DCFH-DA (dichlorofluorescein-diacetate) assay in two different intestinal models [21]. Figure 8 shows the results, expressed as % inhibition of ROS levels released by intestinal cells compared to the positive control, represented by NCM460 cells in which the release of ROS was induced by ABAP, in order to mimic induced oxidative stress. As reported in Figure 8, in this healthy epithelium model, there is no significant difference in ROS release compared to the positive control (Figure 8A). In order to evaluate the potential protective impact, the samples were also tested in the same way in an intestinal model of induced inflammation [24]. Interestingly, Figure 8B shows, for all samples, a protective effect of the inflammatory status through a reduction in the release of ROS content. In particular, sample FM2 (enriched with *Lpb. plantarum* LT100) confirmed a significant reduction compared to the positive control (inflamed NCM460 cells), higher than sample FM3 (enriched by *Lpb. plantarum* C9O4), suggesting a putative positive role of EPSs produced during fermentation in ameliorating inflammation.

## 4. Discussion

Fermented foods and beverages have recently gained attention for their health benefits, due to the presence and direct activity of functional microorganisms, but also to the ability of fermenting microbes to release bioactive metabolites that can have a positive impact for host health [1]. Up to now, there has been emerging interest in exploring the microbial metabolites produced during fermentation, recently defined as post-biotics [42], their relative biological activities, and their potential impact on human health [43]. Among the post-biotics produced during fermentation processes, EPSs have become of considerable interest not only for their rheological and sensorial properties [8], but also for their potential health benefits such as anti-aging, antioxidant, anti-inflammatory, and immunomodulatory effects [9,44]. EPSs produced by LAB, and in particular by lactobacilli, show an interesting structural diversity compared to that associated with other microorganisms. However, the main drawback that limits their exploitation in the food industry is the low production yield, a problem that has not yet been solved to date. EPSs produced by lactobacilli have been well described for their biological and technological role, particularly in dairy products. However, the health effects of EPSs have been discussed similarly to commercial prebiotics in food applications [45]. Recently, in our previous work, we have extensively reviewed the central role of EPSs in fermented foods, especially dairy products, for human health [8]. Among the beneficial effects of EPSs produced by LAB, the potential antioxidant activity has been confirmed by several in vitro and in vivo studies [19,46,47]. It has been reported that a high dose of EPSs (50 mg/kg per day) produced by *Lpb. plantarum* YW11 successfully ameliorated the effects of oxidative stress on an induced aging mouse model [19]. A similar result was also found in a mouse model of aging after the intake of EPS-1 produced by *Lactobacillus helveticus* KLDS1.8701 in which a significantly positive effect on liver injury and oxidative stress was observed, along with a decrease in oxidative stress-related bacteria in the intestinal microbiota, confirming the correlation of the antioxidant activity of EPSs with the modulation of the intestinal microbiota [46]. In this study, we investigated the capability of two strains of *Lpb. plantarum* to differently enhance the antioxidant activity of fermented milks based on their diverse capability (LT100 major producer and C9O4 minor producer) to produce EPSs during the fermentation. Given the scientific evidence related to the ability of some lactobacilli to counteract oxidative stress, with molecular mechanisms not yet fully clarified [48,49], in this study, we wanted to investigate whether EPS was involved in the antioxidant and anti-inflammatory activity of *Lpb. plantarum*. For these reasons, a set of in vitro and ex vivo techniques were applied to determine the antioxidant activity in terms of the direct neutralization of free radicals via hydrogen or electron transfer, ferric reducing power, and the evaluation of anti-inflammatory and antioxidative activity using an intestinal cell model (NCM460) of a healthy epithelium, subjected to both oxidative stress and inflammatory stimuli. Investigations of EPS-producing LAB require an early and rapid in vitro screening, to discriminate bacteria as “ropy or non-ropy” EPS-producing strains. As a preliminary test, a Ruthenium Red plate assay by using different carbon sources (Table S1) allowed us to easily screen strains based on their putative EPSs production (Figure 2). Subsequently, the different EPS production among strains was confirmed using the phenol–sulfuric acid method [26], the most widely used colorimetric assay for calculating EPSs yield [50,51]. Although there could be some interference by other carbohydrates present in the culture medium, colorimetric methods are widely used for a valid quantification of total polysaccharides in food matrices, as previously reported by other authors [10,52,53]. The results confirmed that EPSs production was strictly strain-dependent, as known for many other functional activities [13], and allowed us to select *Lpb. plantarum* LT100 and *Lpb. plantarum* C9O4 as the major (587.8 ± 34.5 mg/L) and minor (115.6 ± 32.5 mg/L) EPSs producers, respectively (Figure 3). It has already been reported that in non-optimized conditions, *Lpb. plantarum* EPSs production ranges from 0.14 to 0.4 g/L [50,54], and in general, the EPS yield is under 1 g/L for most LAB strains [45]. Furthermore, the cross-compatibility test confirmed the absence of any inhibition among the strains (Table S2), an aspect of considerable importance for the success of the fermentation processes and for the effective functionality of the strains when used in a mixture [22]. Indeed, the combination of the two conventional starters with *Lpb. plantarum* strains in the lab-scale production of fermented milks did not affect EPSs production by *Lpb. plantarum* strains, also confirming the capability of LT100 to produce the highest amount of EPSs (465.33 ± 24.27 mg/L) in the final product (Figure 4), whilst C9O4 showed the lowest EPSs content after fermentation (89.03 ± 25.37 mg/L), according to the results obtained in single-inoculated milk fermentation (Figure 3). Reactive oxygen species, such as the superoxide anion (O_2_^−^), hydroxyl radical (OH^•^), nitric oxide (NO), and peroxynitrite (ONOO^−^), can react with all biomacromolecules in cells, causing serious damage and, if accumulated, leading to oxidative stress in vivo, causing many chronic inflammatory conditions in the intestine, a problem of current scientific interest especially in Western society [12,55]. Therefore, the analysis of the antioxidant activity of EPSs was carried out using a combined approach of three different in vitro assays such as ABTS, DPPH, and FRAP, since the molecular mechanisms underlying the antioxidant activity are not yet fully understood [21,56,57]. These assays mimic different reaction environment conditions (hydrophobic for DPPH and hydrophilic for ABTS) and/or different neutralization mechanisms. Thus, using more than one method can overcome some limitations to better assess radical scavenging activity [40]. Some experimental studies reported a negative impact of the digestion process, which leads to a reduction in the antioxidant capacity, for example, of phenolic molecules, as recently shown by Ribeiro and collaborators [58]. Thus, these assays were performed on samples digested following the INFOGEST protocol [32] in order to evaluate the persistence of the antioxidative activity after the digestive process. The results obtained using three different in vitro techniques to determine the antioxidant activity clearly showed that the different production of EPSs by the two *Lpb. plantarum* strains carries a different antioxidant, strictly strain-dependent activity, albeit still high after the digestion process (Figure 6). Overall, for all samples, the ABTS scavenging activity was much higher than DPPH free radical neutralization, as already reported by other studies investigating EPS antioxidant activity due to the hydrophilic nature of EPSs. However, other authors have shown higher values of DPPH scavenging activity (52.23%) by an EPS produced by *Lpb. plantarum* C88 [15,19,41]. Differences in the scavenging rate could be inferred from different experimental aspects such as the concentration and extraction method [59]. The FRAP assay showed significant differences among all samples, with a significant and higher reducing power by yogurt-like samples containing EPSs from *Lpb. plantarum* LT100 (*p* < 0.0001), in accordance with the different ability to produce and release EPSs in the final product (Figure 6C). Furthermore, the comparison of the results obtained with those of a previous study carried out by Prete and collaborators [21] confirms that the EPSs produced by *Lpb. plantarum* have an overall major capacity to neutralize ROSs compared to that of each single strain, highlighting the capability of *Lpb. plantarum* strains to confer and enhance their functionality during fermentation to the final products. In agreement with other studies, our results demonstrate the correlation between greater EPSs production and greater antioxidant activity. A study conducted by Sengül and collaborators showed that a high production of EPSs by *Lb. delbrueckii* ssp. *bulgaricus* B3 corresponded to higher antioxidant and metal ion chelating activities compared to *Lb. delbrueckii* ssp. *bulgaricus* A13, a strain with a lower EPSs production capacity [60]. More recently, Yilmaz and collaborators observed that as the concentration of EPSs produced by each strain increased, in particular, by doubling their quantity, the values of the neutralization activity of hydroxyl radicals increased by approximately double, probably through the capacity of the hydroxyl groups of the EPSs to donate active hydrogen [17]. Other studies have confirmed the antioxidant potential of EPSs produced during milk fermentation through different mechanisms in in vitro systems, by numerous LAB species, including *Lb. delbrueckii* spp. *bulgaricus* [61], *Lacticaseibacillus* (*Lcb.*) *paracasei* ssp. *paracasei*, *Lcb. rhamnosus* [62,63], *Lb. helveticus* [52], and several strains of *Lpb. plantarum* [14,15,64], suggesting the potential use of LAB-derived EPSs as naturally produced antioxidant food additives. Furthermore, it has been reported that some EPSs produced by different LAB species with antioxidant activity have also shown the ability to exert other beneficial effects such as immunomodulatory and anti-inflammatory properties, suggesting a potential correlation in in vitro and in vivo models of chronic intestinal inflammation [17,19,60]. Therefore, in this study, an intestinal epithelial cell model (NCM460) derived from a healthy human colon mucosa [24] was used to evaluate the ROS modulation by samples containing EPSs produced by the two selected *Lpb. plantarum* strains (LT100 and C9O4). The same model was previously used to investigate the potential protective role of *Lpb. plantarum* LT100 and C9O4 cells against oxidative and inflammatory stress, and in mediating the inflammatory response typical of chronic inflammatory bowel diseases (IBDs) [21]. Before the evaluation of the antioxidant and anti-inflammatory capacity of the INFOGEST-digested samples, a non-cytotoxicity test was carried out using the MTT assay, a colorimetric assay widely recognized as an excellent tool for evaluating cell viability [65]. The EPSs produced by the different strains have proven to not inhibit the cell viability of the healthy intestinal epithelium, as shown in Figure 7, demonstrating the non-cytotoxicity of the EPS samples, as previously reported [17]. All the concentrations (5 μL/mL, 10 μL/mL, 20 μL/mL) tested for each sample are likely to leave the cellular metabolic activity of healthy NCM460 cells unchanged or even improved with no significant differences among samples; thus, the highest concentration with no cytotoxic effect was chosen to evaluate ROS modulation. A similar approach has been already reported in testing food-derived samples on other human cell lines [66]. Due to experimental evidence that ROS modulation by LAB and/or food matrices could be influenced by the health status of the intestinal cells, as previously reported [21], ROS modulation by yogurt-like samples was evaluated in a model of a healthy intestinal epithelium subjected to both oxidative stress (ABAP stimulation) and inflammation in order to evaluate the potential protective effect mediated by the modulation of ROS release. To better reflect the actual interaction of EPSs with the biological activity of human cells, ROS modulation was evaluated by carrying out the cellular DCFH-DA assay, a more biologically representative method, widely applied in detecting microbial ROS modulation in different cell lines [67,68,69]. Interestingly, Figure 8 shows the differential impact of fermented milks containing EPSs produced by LAB strains based on the healthy status of the intestinal epithelium, as we have previously obtained by assessing ROS modulation by *Lpb. plantarum* cells [21]. All the samples displayed a preventive effect by eliciting ROS production in healthy cells (Figure 8A), whilst interestingly, in the pathological model of inflammation, a protective effect of the reduction in the inflammatory effect was shown (Figure 8B). ROS are well known as signaling molecules in cell activation [70], and several previous studies reported that probiotics and their metabolites can promote ROS release as a cellular defense mechanism in diverse pathological and inflamed intestinal cells [49,71,72]. During inflammation, ROS can act as second messengers to activate various signaling molecules, such as NF-κB and MAPKs [73,74], involved in the inflammation process. Moreover, the sample FM2 derived from fermentations enriched with *Lpb. plantarum* LT100 showed no significant differences (*p* > 0.05) compared to that of the negative control (untreated healthy cells), suggesting an ability to revert ROS released by inflamed cells to the basal level of healthy cells (Figure 8B). Similar results have been reported by Diao et al. [75] who investigated EPSs from the *Bacillus* sp. strain in a different model of inflammation, speculating that EPSs anti-inflammatory activity through NF-κB inhibition can be mediated by EPSs antioxidant activity and ROS reduction in RAW264.7 macrophages [75]. However, further studies should be needed to investigate more in-depth the precise mechanism of action between ROS modulation and anti-inflammatory activity by EPSs, to confirm the beneficial role of EPSs in ameliorating inflammation via ROS neutralization and modulation, as suggested by our data and other previous studies [40,75].

## 5. Conclusions

Overall, the results highlight a putative role of EPS-producing selected *Lpb. plantarum* to enhance antioxidant and ROS modulation activity in fermented milks, confirming their capability to transfer their functionality during fermentation to the final products. This study also showed a correlation between the EPSs production by the two selected *Lpb. plantarum* strains LT100 and C9O4 and functional activity, with the sample produced with the *Lpb. plantarum* strain (LT100), the highest EPSs producer among tested strains (EPS, 587.77 mg/L), being endowed with a greater antioxidant capacity (ABTS > 99% and FRAP 60.37 ± 1.00 mmol/mL) compared to both a commercial yogurt and the sample produced with the lower-EPS-producer strain (EPS, 115.55 mg/L). Interestingly, the data showed a differential modulation of ROS production by intestinal cells upon either inflamed or oxidative stress with a protective anti-inflammatory effect via a significant reduction in ROS released by yogurt samples enriched with *Lpb. plantarum* strains (FM2: 24.7% and FM3: 17.4% of ROS reduction compared to that of positive control), confirming the same effect previously observed for *Lpb. plantarum* cells [26].

Our data suggest that the use of selected EPS-producing *Lpb. plantarum* strains can be a promising natural strategy to enrich the functionality of fermented milks in terms of ROS modulation and inflammatory-related stress at the intestinal level, representing a promising indication given the current interest in research into the potential benefits of LAB-produced EPSs. However, in vivo studies are still limited, and the production cost and low yield are the limiting factors for the application of different exopolysaccharides at the industrial and biomedical level. The research and selection of high-EPS-producing strains are an ongoing challenging process, as further in vivo and clinical studies are needed to validate the entire healthy potential of EPSs.

## Figures and Tables

**Table 1:** *Lpb. plantarum*, *S. thermophilus*, and *Lb. delbrueckii* subsp. *bulgaricus* investigated in this study.

Species	Strains Code	Origin	Source
*Lpb. plantarum*	O13, C9O4	Table olives	UNITE collection
*Lpb. plantarum*	LT52, LT53 LT100	Raw-milk cheeses	UNITE collection
*S. thermophilus*	DSM20259	Yogurt	UNITE collection
*Lb. delbrueckii* subsp. *bulgaricus*	DSM20081	Bulgarian yogurt	UNITE collection

**Table 2:** Fermented milks produced and investigated in this study.

Samples	Strains
YC	Commercial yogurt
FM1	*S. thermophilus* DSM20259 + *Lb. delbrueckii* subsp. *bulgaricus* DSM20081
FM2	*S. thermophilus* DSM20259 + *Lb. delbrueckii* subsp. *bulgaricus* DSM20081 + *Lpb. plantarum* LT100
FM3	*S. thermophilus* DSM20259 + *Lb. delbrueckii* subsp. *bulgaricus* DSM20081 + *Lpb. plantarum* C9O4

**Table 3:** Microbiological viability and physicochemical characteristics of fermented samples after 24 h of fermentation. Data are reported as mean values ± SEM. Statistical analysis was performed by One-Way ANOVA followed by Tukey’s multiple comparisons post hoc test (different letters mean significant differences among samples, *p* < 0.05).

Physicochemical and Microbiology Characteristic	Yogurt Samples			
	C ^§^	FM1	FM2	FM3
pH	6.89 ± 0.02 ^a^	3.48 ± 0.01 ^b^	3.53 ± 0.01 ^b^	3.42 ± 0.03 ^b,c^
Titratable acidity (g/L of lactic acid)	0.180 ± 0.01 ^a^	1.440 ± 0.01 ^b^	2.160 ± 0.01 ^c^	2.340 ± 0.01 ^d^
Cell viability (Log CFU/g)				
MRS	<LoD *	9.41 ± 0.01 ^a^	9.07 ± 0.02 ^b^	9.09 ± 0.01 ^b^
M17	<LoD *	9.96 ± 0.01 ^a^	9.07 ± 0.01 ^b^	9.14 ± 0.01 ^c^

* LoD: limit of detection (10^1^ CFU/g). ^§^ C represents data from unfermented milk as negative control.

## Data Availability

The original contributions presented in the study are included in the article and supplementary material, further inquiries can be directed to the corresponding author.

## Supplementary Materials

The following supporting information can be downloaded at https://www.mdpi.com/article/10.3390/foods13111663/s1. Figure S1: Glucose calibration curve used for EPS quantification (0–750 mg/L); Figure S2: Evaluation of viability of human intestinal cells incubated with undigested fermented milks at different concentrations. Table S1: Different carbon sources used in the Ruthenium Red assay; Table S2: Growth compatibility assay in which plus (+) means inhibition and minus (−) means growth among all strains.

## Abbreviations

EPSs: exopolysaccharides; DPPH: 1,1-diphenyl-2-picrylhydrazyl radical; ABTS: 2,2-azino-bis (3-ethylbenzothia-zoline-6-sulfonic acid); FRAP: ferric reducing antioxidant power; ROS: Reactive Oxygen Species; EFSA: European Food Safety Authority; LAB: lactic acid bacteria; NCM460: Normal Colonic Mucosa Cells; DSM: Leibniz Institute DSMZ-German Collection of Microorganisms and Cell Cultures; MRS: de Man–Rogosa–Sharpe; PCA: Plate Count Agar; RR: Ruthenium Red; UHT: Ultra-High Temperature; TCA: Trichloroacetic Acid; NaOH: sodium hydroxide; FBS: Fetal Bovine Serum; Pen-Strep: Penicillin–Streptomycin; NEAAs: Non-Essential Amino Acids; MTT: 3-(4,5-dimethylthiazole-2-yl)-2,5-phenyl tetrazolium bromide; DMEM: Dulbecco’s Modified Eagle’s Medium; OD: Optical Density; DCFH-DA/DCF-DA: Dichlorofluorescein Diacetate; HBSS: Hanks’ Balanced Salt Solution; ABAP: 2,2’-azobis (2-amidinopropane) dihydrochloride solution; TNF-α: Tumor Necrosis Factor-alpha; IFN-*γ*: Interferon-gamma; IL1-β: Interleukin 1-beta; IBD: Inflammatory Bowel Disease; NF-kB: Nuclear Factor kappa-light-chain-enhancer of activated B cells; MAPKs: Mitogen-Activated Protein Kinases; SEM: Standard Error of Mean.
