# Supplementary material for: Improving the Antioxidant and Anti-Inflammatory Activity of Fermented Milks with Exopolysaccharides-Producing Lactiplantibacillus plantarum Strains
Source: Foods. 2024 May 25;13(11):1663. doi: 10.3390/foods13111663 (PMC11171883; doi:10.3390/foods13111663)

Table S1. Different carbon sources used in the Ruthenium Red assay

Media	Glucose (g/l)	Sucrose (g/l)
**MRS**	20	-
**MRS + sucrose**	20	10
**MRS w/o glucose + sucrose**	-	20
**PCA**	20	-
**PCA + sucrose**	20	10

Table S2. Growth compatibility assay in which plus (+) means inhibition and minus (-) means growth among all strains.

Donor strain	C9O4	LT52	LT53	O13	LT100	DMSZ20259	DMSZ20081
**C9O4**	-	-	-	-	-	-	-
**LT52**	-	-	-	-	-	-	-
**LT53**	-	-	-	-	-	-	-
**O13**	-	-	-	-	-	-	-
**LT100**	-	-	-	-	-	-	-
**DSM20259**	-	-	-	-	-	-	-
**DSM20081**	-	-	-	-	-	-	-

Figure S1. Glucose calibration curve used for EPS quantification (0-750 mg/L).

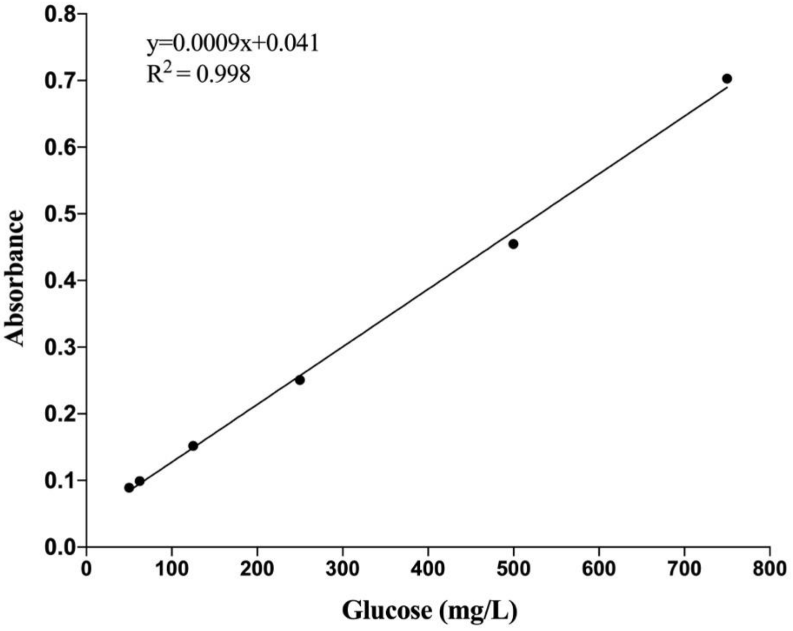

Figure S2. Evaluation of viability of human intestinal cells incubated with undigested fermented milks at different concentrations: A) 5 μL/mL, B) 10 μL/mL and C) 20 μL/mL Data are reported as mean values ± SEM and they were statistically analyzed using One-Way Anova followed by Tukey's multiple comparisons test ($p > 0.05$).

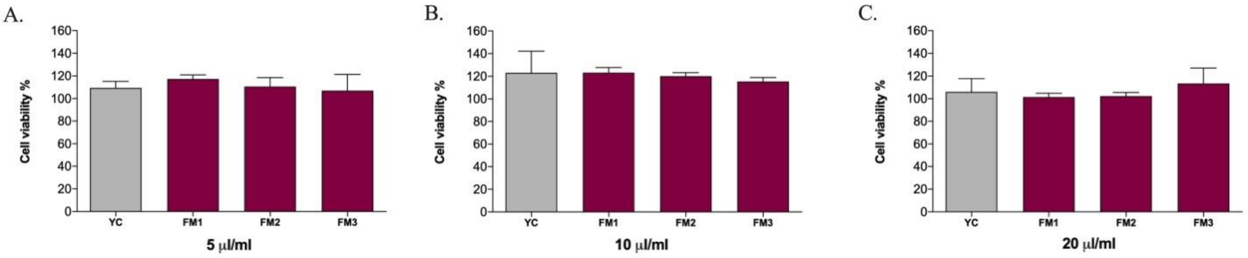

Supplement: Supplementary file 1 [file foods-13-01663-s001.zip › foods-2982898-supplementary.pdf]
